# Increased pulse wave transit time after percutaneous coronary intervention procedure in CAD patients

**DOI:** 10.1038/s41598-017-18520-6

**Published:** 2018-01-08

**Authors:** Lizhen Ji, Chengyu Liu, Peng Li, Xinpei Wang, Changchun Liu, Yinglong Hou

**Affiliations:** 1 School of Control Science and Engineering, Shandong University, Jinan, 250061 China; 2 Institute of Environment and Ecology, College of Geography and Environment, Shandong Normal University, Jinan, 250014 China; 3 Department of Cardiology, Shandong Provincial Qianfoshan Hospital, Shandong University, Jinan, 250014 China

## Abstract

Pulse wave transit time (PWTT) has been widely used as an index in assessing arterial stiffness. Percutaneous coronary intervention (PCI) is usually applied to the treatment of coronary artery disease (CAD). Research on the changes in PWTT caused by PCI is helpful for understanding the impact of the PCI procedure. In addition, effects of stent sites and access sites on the changes in PWTT have not been explored. Consequently, this study aimed to provide this information. The results showed that PWTT significantly increased after PCI (*p* < 0.01) while the standard deviation (SD) of PWTT time series had no statistically significant changes (*p* = 0.60) between before and after PCI. Significantly increased PWTT was found in the radial access group (*p* < 0.01), while there were no significant changes in the femoral access group (*p* > 0.4). Additionally, PWTT in the left anterior descending (LAD) group significantly increased after PCI (*p* < 0.01), but the increase that was found in the right coronary artery (RCA) group was not significant (*p* > 0.1). Our study indicates that arterial elasticity and left ventricular functions can benefit from a successful PCI procedure, and the increase of peripheral PWTT after PCI can help to better understand the effectiveness of the procedure.

## Introduction

Pulse wave transit time (PWTT) refers to the time interval that a pulse wave propagates through the distance between two arterial sites^1,2^. It is the time interval from the electrocardiogram (ECG) R-wave peak to the foot of the synchronized photoplethysmography (PPG) signal^3^. Regardless of the resting or exercise state^4,5^, an increase in PWTT generally indicates a decrease in arterial stiffness, which is often associated with cardiovascular events^6,7^. As one of the most common cardiovascular diseases, coronary artery disease (CAD) often requires treatment by percutaneous coronary intervention (PCI), and the demand for PCI has increased tremendously. Bundhun *et al*. have found that there are no significant differences in adverse outcomes between same day discharge and overnight hospital stay^8^. Therefore, a better explanation of the changes in PWTT of CAD patients, especially between before and after PCI, is helpful to understand the effectiveness of PCI. Significantly lower carotid-femoral aortic PWV after PCI has been reported^9^, but there is insufficient literature discussing peripheral PWTT. In this study, we explore the changes of peripheral PWTT within 24 h after a PCI procedure.

PCI can be carried out through radial access or femoral access; the safety and effectiveness of these procedures have been assessed in numerous studies^10–14^. In 2012, Bertrand *et al*. compiled a meta-analysis of studies regarding PCI performed via radial access or femoral access. The results suggest that the procedure performed through radial access has better outcomes with respect to complications, bleeding, and death^13^. Hannan *et al*.^14^ also found a trend of mortality reduction for patients treated through radial access, though the difference was not significant. Consequently, this study aims to explore the influence of the different artery access points on the changes in PWTT between before and after the PCI procedure.

The left anterior descending (LAD) artery and the right coronary artery (RCA) are two main sites for performing PCI^15^. Previous studies found good outcomes in patients with revascularization in isolated LAD^16,17^. It is known that the LAD is approximately twice as large as the RCA^18,19^. Here, we investigate if stents in the isolated LAD or RCA can influence changes in peripheral PWTT and whether there will be different effects for these cases.

## Results

### PWTT differences between before and after PCI

Table 1 shows the results of PWTT and standard deviation (SD) of PWTT time series, along with SBP from before and after PCI for each patient. Statistical results showed that PWTT significantly increased after PCI (210 ± 4 ms vs. before 204 ± 3 ms, *p* < 0.01) while the SD of PWTT and SBP revealed no significant difference (before 4.09 ± 0.35 ms vs. after 4.17 ± 0.35 ms, *p* = 0.60; before 127 ± 2 mmHg vs. after 125 ± 3 mmHg, *p* = 0.18).

**Table 1:** Results of PWTT, SD of PWTT and SBP from before and after PCI for all patients.

Subject No.	PWTT (ms)		SD of PWTT (ms)		SBP (mmHg)	
	Before PCI	After PCI	Before PCI	After PCI	Before PCI	After PCI
1	194	206	3.81	3.61	150	150
2	215	226	3.04	3.76	137	131
3	202	193	6.89	3.72	117	118
4	185	201	4.41	6.22	140	138
5	200	205	4.18	3.91	130	130
6	182	196	3.34	2.86	117	117
7	220	243	7.31	6.93	140	143
8	197	222	4.00	4.13	134	126
9	201	219	2.33	3.73	124	126
10	169	178	1.96	2.52	115	110
11	192	208	2.59	5.18	130	139
12	190	200	1.62	3.21	121	126
13	207	203	2.64	1.67	125	129
14	217	209	6.68	8.76	125	117
15	195	189	2.91	2.06	136	132
16	217	217	5.83	7.28	130	135
17	216	207	7.21	3.09	136	131
18	207	211	2.98	2.69	119	115
19	219	229	4.51	3.99	114	107
20	252	269	7.66	6.72	121	117
21	209	205	3.72	4.46	135	138
22	180	177	3.51	3.43	143	148
23	198	201	2.86	2.82	130	130
24	196	208	4.46	5.32	129	130
25	201	195	1.57	2.26	133	126
26	204	216	2.43	2.32	117	115
27	230	234	5.97	5.89	127	108
Mean	204	210	4.09	4.17	129	127
SE	3	4	0.35	0.35	2	2
*p*	<0.01		0.60		0.18	

### Effects of access sites and stent sites on PWTT

**Figure 1:**
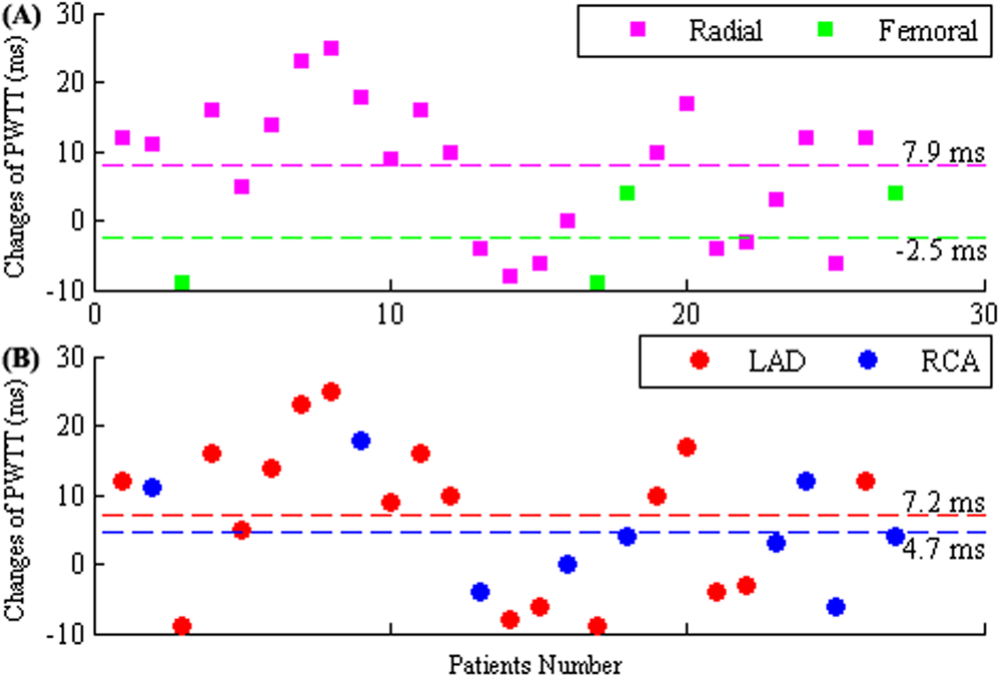
Scatter plots of the changes in PWTT in radial access group and femoral access group (**A**), as well as in LAD and RCA groups (**B**). Pink squares represent cases of patients in radial access group and green squares denote cases in femoral access group in section (**A**). In section (**B**), the red plots represent values of patients in LAD and the blue plots denote values in RCA. Mean changes of all four groups are shown by the dotted lines with corresponding color.

The changes in PWTT (value after PCI minus value before PCI) are shown in Fig. 1 with specific annotation for the radial and femoral access sites (A) and for the LAD and RCA stent sites (B). As shown in Fig. 1(A), the majority of patients (85%) underwent PCI through radial access, and among these, sixteen patients exhibited increased PWTT, six had decreased PWTT, and one had no change after the PCI procedure. Four patients (15%) underwent the procedure through femoral access when the radial access failed, and among these patients, two had increased PWTT while the others had decreased PWTT after the procedure. The mean changed value was 7.9 ms for the radial access group, and −2.5 ms for the femoral access group. Eighteen patients accepted stent implantation in the LAD, which was approximately 67% of the total patients, and the other nine patients (approximately 33%) accepted stent implantation in the RCA, as shown in Fig. 1(B). In the LAD group, twelve patients exhibited an increased PWTT after PCI, while six patients had a decrease. The mean value of PWTT change was 7.2 ms. In the RCA group, six patients exhibited an increase, one remained unchanged and the other two had a decrease in the PWTT, resulting in a mean change of 4.7 ms.

**Figure 2:**
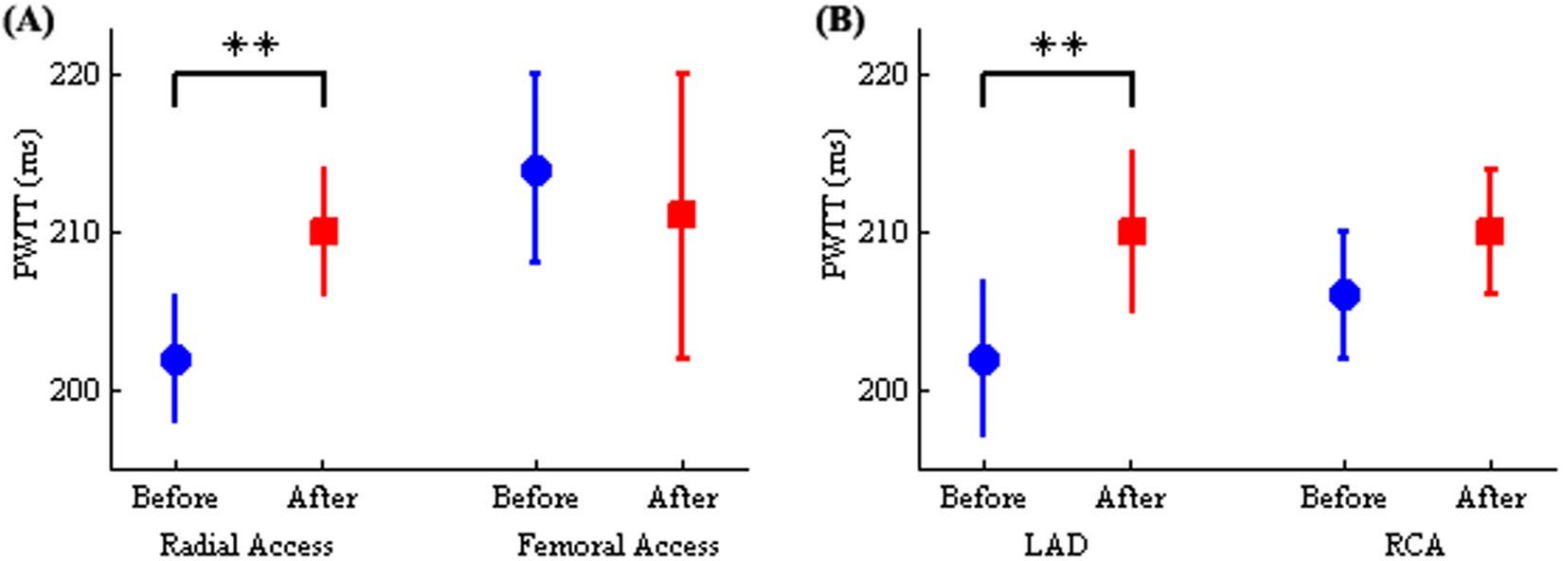
Effects of access sites and stent sites on PWTT in CAD patients. Influence of PCI from radial and femoral access sites (**A**), as well as with stent sites in LAD and RCA (**B**) on PWTT interval for CAD patients. **denotes *p* < 0.01.

Figure 2(A) shows the results of PWTT in both groups divided by access site, before and after the PCI procedure. PWTT in the radial access group after PCI significantly increased (210 ± 4 ms vs. before 202 ± 4 ms, *p* < 0.01), while a slight decrease was found in the femoral access group (211 ± 9 ms vs. before 214 ± 6 ms, *p* > 0.4), though there was no significant difference between before and after PCI.

PWTT before and after PCI are shown for both the LAD and RCA groups in Fig. 2(B). The results show that PWTT in the LAD group after PCI significantly increased (210 ± 5 ms vs. before 202 ± 5 ms, *p* < 0.01). While there was not a significant difference between the two procedures, there was only a slight increase in the PWTT (before 206 ± 4 ms vs. after 210 ± 4 ms, *p* > 0.1) for the RCA group.

## Discussion

This study investigated the changes in PWTT within 24 h after PCI in CAD patients, and a significant increase in PWTT was observed. We also explored the effect of access and stent sites on the changes in PWTT. As a result, in the case of radial access and the LAD stent site, a significant increase in PWTT after PCI was observed. On the other hand, when the femoral access was chosen or the RCA stent implantation was performed, there were no significant changes in PWTT after PCI.

Kalay *et al*.^9^ have shown that carotid-femoral aortic PWV after PCI was significantly lower than before the procedure, which has been associated with a decrease in artery stiffness levels. Furthermore, Lang *et al*. and Tavil *et al*.^20,21^ have found that the decrease in artery stiffness is closely correlated with the enhancement of left ventricular functions. This could be explained because improved left ventricular function can increase the cardiac output, inhibiting the activity of the sympathetic nerve, and reducing tension of the artery while improving its elasticity. In this study, we found a significantly increased peripheral PWTT shortly after PCI, which indicates that PCI is helpful for improving arterial elasticity and the left ventricular functions. Other studies have shown that successful PCI should be able to enhance the left ventricular functions shortly after the procedure^20,22^. Thus, the increase of PWTT after the procedure can also be considered as an indicator of a successful PCI, and is measured by a noninvasive method. In addition, it has been reported that there was no significant difference in ambulatory blood pressure between 3–6 days after PCI and 1–3 days before PCI^23^. Our study also found that SBP did not significantly decrease within 24 h after PCI. The autonomic regulatory function and regulation mechanisms that perform blood pressure control functions have not yet been restored in the short period after PCI (e.g., 24 h or 3–6 days); therefore, the balance in blood pressure cannot be recovered and retained to its preoperative value by this time.

It has been reported that compared with femoral access during PCI^13,24,25^, radial access has a stronger association with reduced adverse cardiovascular events. Guidelines from Europe^26^ have also endorsed the preferential use of radial access. In this study, PWTT significantly increased in the radial access group after PCI, whereas it displayed a decreasing trend in the femoral access group. These results indicate that radial access during PCI generated better outcomes than femoral access, which is in accordance with previous studies.

O’Keefe *et al*.^16^ and Holmes *et al*.^17^ suggested that patients with isolated LAD disease generally had positive revascularization results such as low in-hospital adverse event rates and good long-term outcomes. Our results show that patients with isolated LAD stents had significantly increased PWTT, while patients in the RCA group did not see an increase, which suggests that, within the short term after PCI, the artery status and left ventricular function of patients in the LAD group were improved compared to those in the RCA group. This might be attributed to the fact that the LAD supplies a large amount of the myocardium and is in close proximity to the distal left main coronary artery^17^, thus, revascularization here would contribute to the majority of the myocardium blood supply and improve the cardiovascular function. Additionally, Cortigiani *et al*.^27^ found that abnormal coronary flow reserve in the LAD had a higher annual hard event rate than abnormal coronary flow reserve in the RCA, which partially supports our results. Nevertheless, Kalay *et al*.^9^ reported that stents, both in the LAD and the RCA, brought about a significant decrease in PWV, which is not entirely consistent with our results. This might be due to our different measuring sites; the status of the aortas might be preferentially improved compared with the peripheral artery due to their closer proximity to the coronary artery.

This study investigated the influence of PCI on the PWTT. Our results showed a significantly increased PWTT after the procedure, which indicated improved cardiovascular function and a successful PCI procedure. In addition, the influence of different stent and access sites on the PWTT was explored. The results suggested that radial access had better outcomes than femoral access, and revascularization in the LAD performed better than in the RCA. Nevertheless, the sample size of this study was relatively small, especially in the femoral access group and RCA group, and a further large-scale study is needed.

## Methods

### Subjects

Twenty-seven patients with coronary artery stenosis (aged between 44 and 80) were enrolled in this study. Routine ECG and echocardiography examinations were performed. Subjects who had frequent ectopic beats and left ventricular ejection fraction less than 50% were eliminated before participation. Patients were confirmed by coronary angiography, and all had at least one main coronary branch stenosed for over 50%. Coronary angioplasty was performed according to established PCI standards. This clinical trial follows the declaration of Helsinki and regulations of China. CAD patients only were selected for this study; we did not include patients with other diseases such as diabetes mellitus, heart failure, etc. The study obtained full approval from the Institutional Review Board of Shandong Provincial Qianfoshan Hospital, and informed consent was required for each patient before participation. Basic characteristics of the patients are shown in Table 2.

**Table 2:** Basic characteristics of all enrolled CAD patients.

Variables	Value	Range
Number (M/F)	27 (15/12)	—
Age (year)	62 ± 9	44–80
Height (cm)	168 ± 8	150–181
Weight (kg)	72 ± 12	42–91
BMI (kg/m^2^)	25.3 ± 2.9	20.8–28.7
SBP (mmHg)	129 ± 9	114–150
DBP (mmHg)	79 ± 8	63–93
HR (beat/min)	63 ± 7	50–77

Note: value is expressed as number (male/female) or mean ± standard deviation (SD). BMI: body mass index, SBP: systolic blood pressure, DBP: diastolic blood pressure, HR: heart rate.

### Protocol

Measurements were taken in a quiet and temperature controlled room (25 ± 3 °C) at Shandong Provincial Qianfoshan Hospital, Shandong University, using a cardiovascular function detection device (CV FD-II, inspection report number: D2012040606), which was produced by Huiyironggong Tech. Co., Ltd. (Jinan, China). Before commencing signal recording, each patient lay supine on a measurement bed for 10 minutes rest to allow for cardiovascular system stabilization. ECG electrodes were attached to the right wrist and to the right and left ankles to acquire a standard limb lead-II ECG. A photoelectric sensor was attached to the tip of the left forefinger to acquire finger PPG waves. The ECG and PPG signals were synchronously recorded at a sampling frequency of 1 kHz for 5 min before and after the PCI procedure. Patients were told to breathe regularly and gently during the measurements. Blood pressure measurement was carried out with an arm blood pressure monitor after signal acquisition. The measurement was performed within 24 h before and after PCI by the same operator.

### PWTT series construction

**Figure 3:**
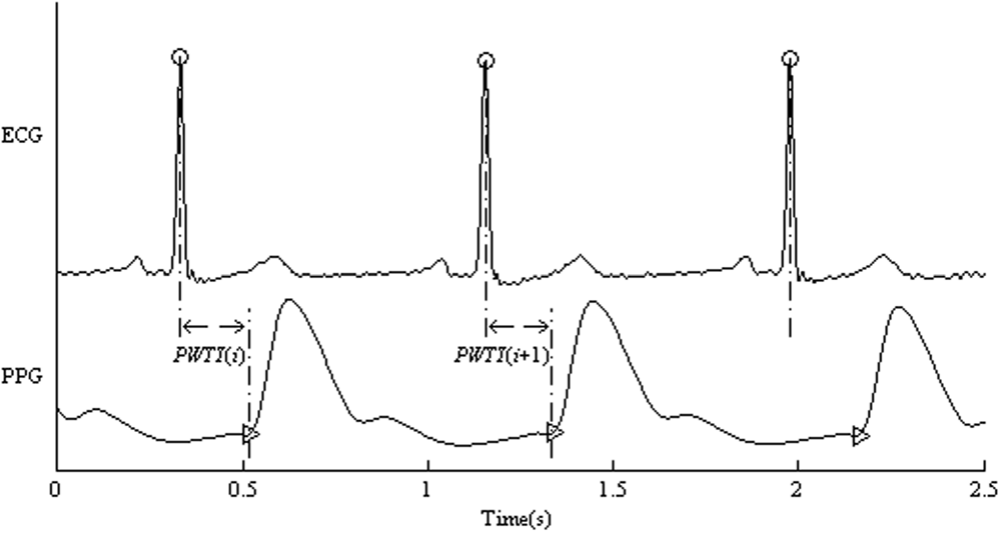
Construction process of PWTT time series from a segment of synchronously recorded ECG and PPG signals. “” denotes the R-wave peak and “” denotes the foot of PPG. PWTT is defined as the interval from the R-wave peak to the foot of PPG signal in the same heart cycle.

Slow varying components (0–0.05 Hz) in ECG and PPG signals were initially removed. ECG R-wave peaks were detected by a template-matching procedure^28^. With the location of R-wave peaks, the corresponding systolic feet were detected from the first-order differential signal of PPG^29^. PWTT series were constructed using the means of all intervals from the R-wave peaks to the feet of the corresponding PPG pulses in the same heart cycle^3,30^. Figure 3 demonstrates the construction process of PWTT time series from the synchronously recorded ECG and PPG signals. Anomalous intervals due to ectopic beats or poor signal quality were visually identified and removed from the PWTT time series.

**Figure 4:**
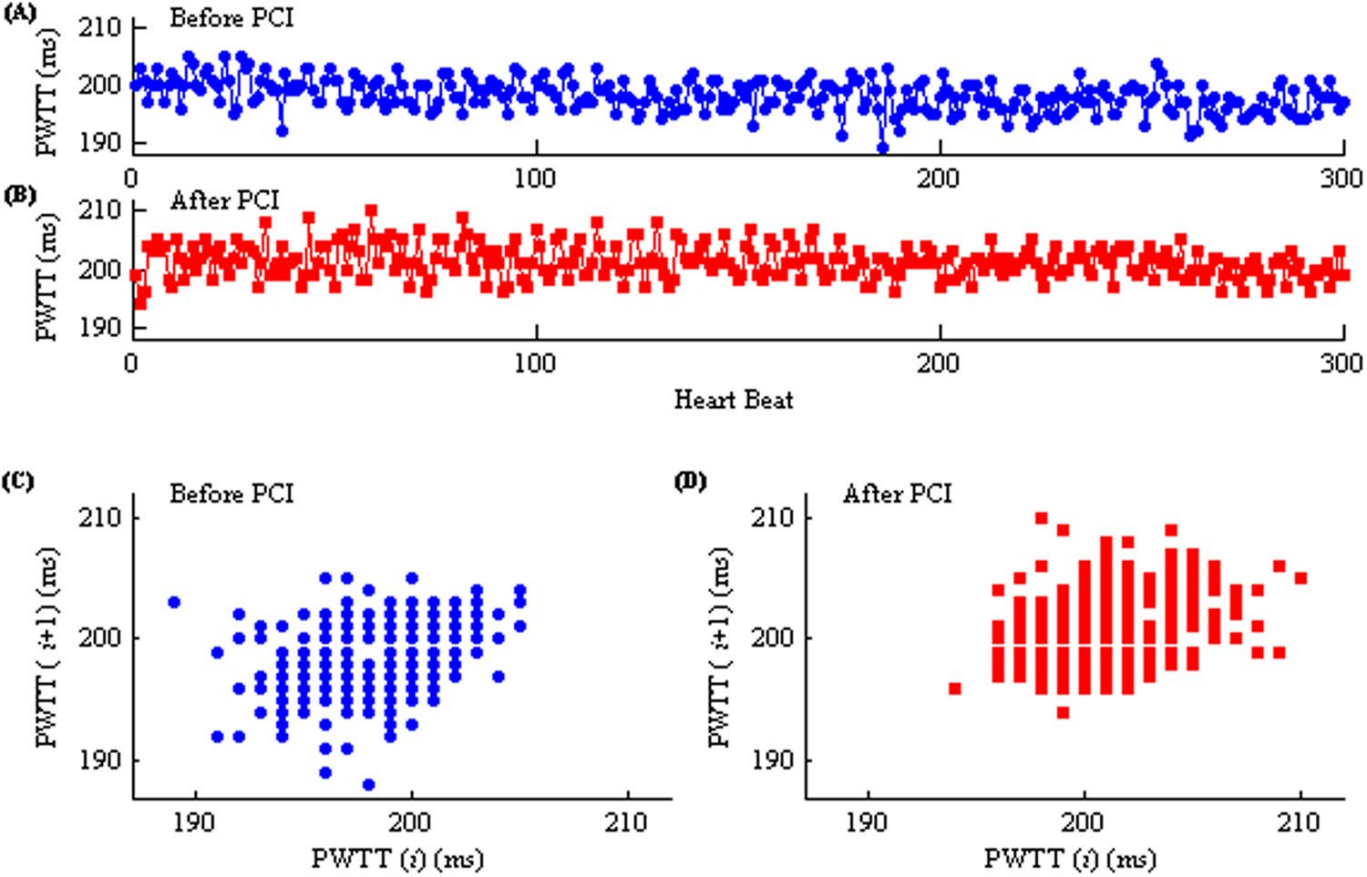
Examples of the PWTT interval series before (**A**) and after (**B**) PCI from one patient. Section (**C**) and (**D**) show the corresponding scatter plots of the two PWTT series.

Figure 4 shows examples of the PWTT series from before (A) and after (B) PCI procedures, as well as the corresponding scatter plots (C and D) for the above-mentioned two PWTT series, respectively. The scatter plots in Fig. 4 section (D) are closer to the upper right than in section (C), which indicates that the values of beat-to-beat PWTT after PCI are more concentrated and higher than before PCI.

### Statistical analysis

**Figure 5:**
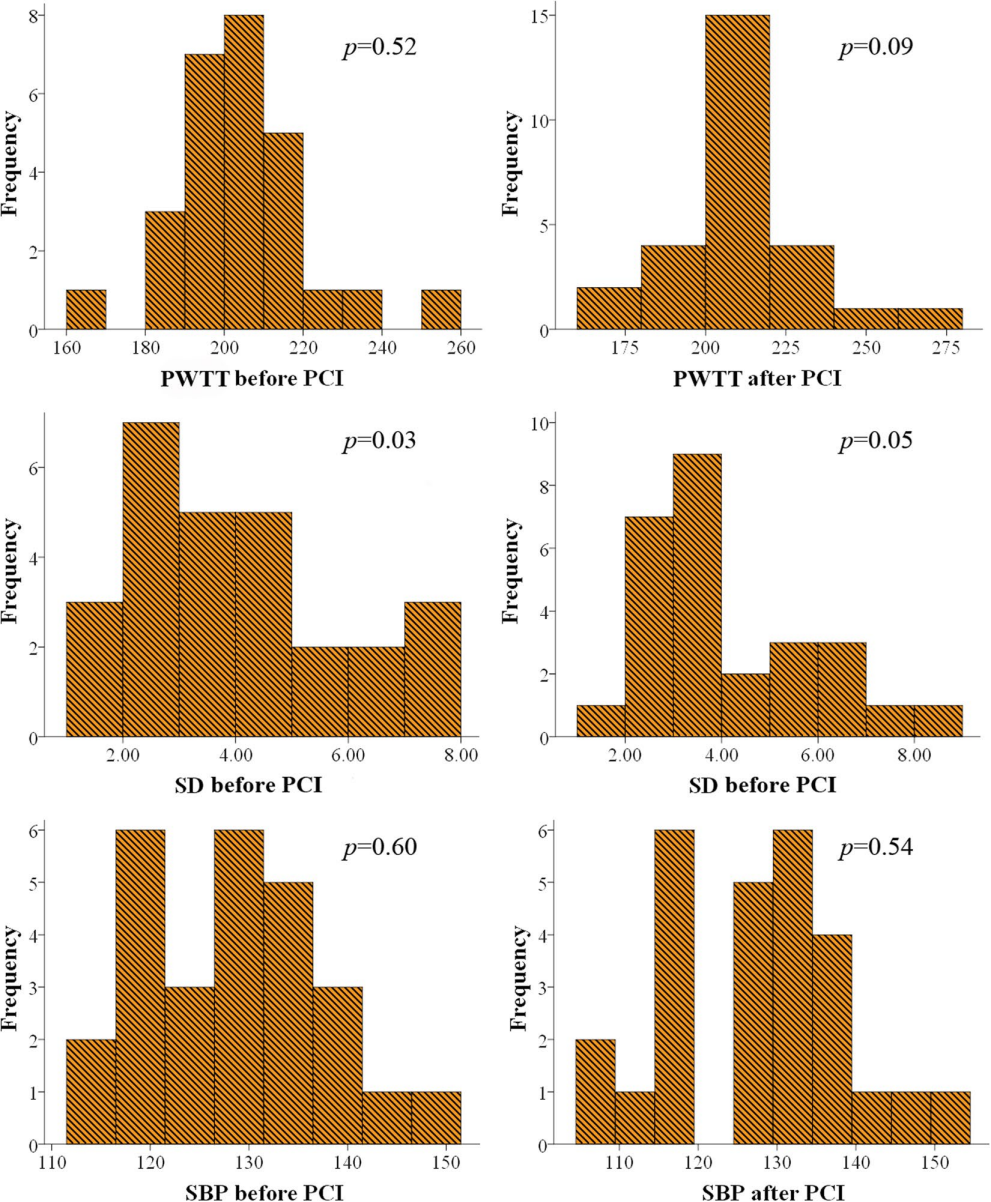
Results of normal distribution test of PWTT, SD and SBP before and after PCI.

The individual mean PWTT value (PWTT) and standard deviation (SD) were calculated separately from the PWTT time series before and after PCI. The above calculations did not follow normal distributions (all *p* < 0.05/6 using the Bonferroni correction), according to the results of the Shapiro-Wilk test which are shown in Fig. 5. Therefore, the Wilcoxon non-parametric test was used to verify significant differences of the PWTT and SD results, as well as SBP between two measurements, before and after the PCI procedure. Furthermore, patients were divided into two groups according to the stent site (i.e., LAD group and RCA group), who were also categorized in terms of the access site (i.e., radial access group and femoral access group). The significant differences between the two groups in each type were analyzed to determine the effects of stent site and access site on the changes of PWTT. Statistical analyses were performed using the SPSS software (Ver. 20, IBM, USA). Statistical significance was accepted at *p* < 0.05.

### Data availability

All data generated or analyzed during this study are included in this published article.
